# Items

**Published:** 1904-03

**Authors:** 


### ITEMS.

---

THE Central Institute of Physical Therapeutics, at Rome, Italy, has published a handsome brochure of 81 pages, which gives a detailed account of the conduct of the institution from the date of its establishment to the present time. It appears, according to this booklet, that it was inauguarated May 8, 1902, in the presence of the authorities, distinguished personages, and a large gathering of citizens. It is one of the largest, best built, and most completely equipped institutions of its kind in the world. Professor Charles Colombo is the director of the institute and since it was opened he and his assistants have treated over 7,000 persons, 74 per cent. of whom have recovered. The pamphlet is profusely illustrated and contains much interesting information.

---

*Facetiae Medicorum* is the title of a brochure recently issued by the New York Pharmacal Association, Yonkers, N. Y. It contains a collection of the wit and humor of medicine in prose, poem, and picture, gleaned from various sources, selected and reprinted from the files of the *Doctor's Factotum.* It will be sent to any physician on application to the publishers.

---

A HANDSOME lithograph in many colors of a Medicine Man of the Sioux Indians has been sent to every physician in the United States by the proprietors of the tongaline preparations and ponca compound. Any physician who has not received this unique and artistic reproduction of a famous Indian Chief can obtain it by writing to the Mellier Drug Company, Saint Louis.

